# Predictors of Dental Misconceptions Among Taif Population, Saudi Arabia

**DOI:** 10.3290/j.ohpd.c_1812

**Published:** 2025-01-15

**Authors:** Shaimaa Al Harthi

**Affiliations:** a Shaimaa Al Harthi Assistant Professor, Department of Oral and Maxillofacial Surgery and Diagnostic Sciences, Faculty of Dentistry, Taif University, Saudi Arabia.

**Keywords:** demographic factors, health education, myths and misconceptions, oral health

## Abstract

**Purpose:**

Health education programmes play a crucial role in enhancing oral health literacy and improving treatment outcomes. However, myths and misconceptions about oral health are widespread, affecting individuals’ behaviours and their willingness to seek appropriate treatment. This study aimed to investigate the prevalence of oral health myths and misconceptions among adults in Taif, Saudi Arabia, and to explore potential associations with demographic factors.

**Materials and Methods:**

Participants from community health programmes in Taif (March–June 2024) completed a Google Forms questionnaire on dental myths. Eligibility criteria: adults (≥18 years) without cognitive, hearing, or vision impairments. The questionnaire included sociodemographic details and 22 myth-related questions. Data analysis involved descriptive statistics and multiple linear regression using the Statistical Package for Social Sciences (SPSS), with significance set P ≤ 0.05.

**Results:**

The study included 429 participants. Knowledge of dental myths was highest in the ‘Dental Treatment’ domain (mean score: 6.42), followed by ‘Oral Hygiene Practices’ (3.46) and ‘Deciduous Teeth and Pregnancy’ (3.48). Higher education and healthcare-related majors were statistically significantly associated with better knowledge. Conversely, males and older age groups had lower knowledge scores.

**Conclusion:**

This study highlights the prevalence of dental myths in Taif and their association with demographic factors. Higher education- and healthcare-related fields correlate with better knowledge, but statistically significantly gaps remain, particularly among the less educated, certain occupational groups, males, and single individuals. Targeted educational interventions are essential to improving dental health knowledge and practices and enhancing oral health outcomes in the community.

Myths are defined as prevalent beliefs, misconceptions, or false understandings that are widely accepted within a cultural context but lack any basis in truth.^2,18^ Despite significant advancements in medical and dental sciences, oral health conditions remain globally neglected, resulting in substantial social and economic burdens.^2,18^ This neglect is exacerbated by culturally transmitted beliefs that perpetuate collective misunderstandings of oral health.^8,21,24^ These persistent misconceptions are rooted in various factors, including limited education, cultural traditions, and social misunderstandings, necessitating targeted interventions to dispel them.^17^

Cultural influences also shape patients’ perspectives on oral health, often leading to reliance on traditional remedies or avoidance of professional dental care owing to superstitions.^7,10,11^ Such misconceptions contribute not only to dental anxiety but also to the reluctance to seek necessary treatments, thereby compromising overall oral health.^1^ This issue is particularly pertinent in Saudi Arabia,^3,13,19^ a developing nation that is currently ranked 40th on the United Nations Human Development Index. The country boasts an adult literacy rate of 97.59% for individuals aged 15 and older, with 80.9% of males and 71.3% of females aged 25 and older having received at least some secondary school education.^20^ Despite these educational achievements, misconceptions about oral health persist, underscoring the need for targeted interventions.

Effectively addressing these myths necessitates a multifaceted approach involving patient education, evidence-based communication strategies, and culturally sensitive interventions.^7^ Dental professionals play a pivotal role in dispelling these myths, providing accurate information, and fostering positive attitudes toward oral health.^5,19^ Therefore, the aim of this study was to investigate the prevalence of common myths and misconceptions regarding oral health among adults in the community of Taif, Saudi Arabia. Furthermore, this study sought to explore any associations between the prevalence of these myths and specific demographic factors. By identifying how these misconceptions are distributed across various demographic segments, this study aims to provide targeted insights that can enhance the effectiveness of public health interventions and educational campaigns.

## MATERIAL AND METHODS

Participants were recruited from community health programme attendees held in public venues across the city of Taif between March and June 2024. Eligible participants were aged 18 years or older, free from cognitive, hearing, or vision impairments, and willing to participate voluntarily. No specific exclusion criteria were applied. Surveys and questionnaires distributed via Google Forms were used to assess knowledge of common dental misconceptions. The questionnaire required approximately 10–15 minutes to complete. Participation was entirely voluntary.

The questionnaire consisted of two sections: Section A gathered detailed sociodemographic information to explore how different demographic factors might influence beliefs in dental myths. Participants provided information on their sex (female or male), age, marital status, level of education, occupation, and study major. Age was categorised into generational cohorts: Generation Z (18–27 years), Generation Y/Millennials (28–43 years), Generation X (44–59 years), and Baby Boomers (≥ 60 years), to explore potential generational differences in beliefs. Marital status options included married, single, and divorced. The participants’ education levels ranged from no formal education to doctorate degrees. Occupation categories included students, governmental and private sector employees, freelancers, retirees, and the unemployed. Study majors were classified into various fields: Medicine and Healthcare; STEM; Business and Administration; Law; Arts and Creative Industries; Humanities and Social Sciences; Non-profit and Social Services; and other areas. Section B comprised 22 self-administered closed-ended questions categorised into three domains: ‘Oral Hygiene Practice’, ‘Dental Treatment’, and ‘Deciduous Teeth and Pregnancy’. The questions were meticulously crafted based on a thorough review of existing literature on dental myths and misconceptions prevalent in both global contexts and within Saudi Arabia. Key studies^2,3,13^ were identified that highlighted specific myths related to oral hygiene practices, dental treatments, and beliefs concerning deciduous teeth and pregnancy. The literature review revealed that certain misconceptions were particularly pervasive in the region, such as beliefs about the harms of dental treatments during pregnancy or misconceptions about oral hygiene practices. By incorporating these findings, the questionnaire addressed both universal and region-specific misconceptions, ensuring it was comprehensive and culturally relevant. The questionnaire was initially drafted in English and translated into Arabic to ensure clarity and cultural appropriateness for the target population. The translation and adaptation process followed the World Health Organization’s guidelines for instrument translation and adaptation. Validation of the questionnaire involved administering it to 10 subjects at the University Dental Hospital clinics who were excluded from the main analysis. The same subjects completed the questionnaire again after a two-week interval for test-retest reliability assessment. Any ambiguities or misunderstandings identified during validation were addressed, and necessary adjustments were made to enhance clarity and reliability.

### Ethical Considerations

This study was conducted in accordance with the Declaration of Helsinki and received approval from The Taif University Scientific Research Ethics Committee (approval number: 45–280). Informed consent was obtained from all participants prior to their involvement in the study. Measures were implemented to ensure the confidentiality and privacy of participant information throughout the study duration.

### Statistical Analysis

The sample size for this study was determined using a 95% confidence level and a 5% margin of error, with an estimated prevalence of 50%. Initially, this yielded a sample size of 385 participants, which remained unchanged after applying the finite population correction, ensuring reliable statistical precision for the study. Descriptive statistics were computed for all the variables, including frequencies and percentages, as indicated. Adjusted multiple linear regression analysis was performed to explore the effect of the different demographic parameters on the average knowledge of common dental myths in all three domains. Categories with few numbers were collapsed as needed. The results are presented as adjusted beta coefficients and relevant 95% confidence intervals (CIs). The normality of the data was assessed using the Kolmogorov–Smirnov test and histogram plots, while the homogeneity of variances was verified using residual plots. In all the models, the variance inflation factor (VIF) was used to detect any problems related to multicollinearity among the variables, while Cook’s distance was used to identify any substantial outliers. No violations were detected regarding these assumptions in any of the models. Additionally, the goodness-of-fit for each model was evaluated using the adjusted coefficient of determination (Adj. R^2^). All analyses were performed using the Statistical Package for Social Sciences (SPSS) software (Version 26.0, Chicago, IL, USA). A P value of ≤ 0.05 indicated statistical significance for all two-sided statistical tests.

## RESULTS

Overall, the study included a total of 429 participants. The characteristics of the participants are summarised in Table 1. The sample consisted of 53.1% females and 46.9% males. The age distribution was as follows: Generation Z (18–27 years) comprised 25.6%, Generation Y/Millennials (28–43 years) 41.0%, Generation X (44–59 years) 22.1%, and Baby Boomers (≥ 60 years) 11.2%. Most participants held a bachelor’s degree (55.7%), followed by high school graduates (19.6%). A small percentage had only a primary school education (0.9%). Regarding occupation, most participants were employed in the governmental sector (46.6%), followed by retirees (11.0%) and students (7.9%). A significant portion of the participants were married (68.5%), 26.6% were single, and 4.9% were divorced. The most common fields of study were business and administration/law and legal studies (14.9%), followed by medicine and healthcare (11.2%).

**Table 1:** Characteristics of the study participants (N = 429)

Characteristic	n (%)
Sex Females Males	228 (53.1) 201 (46.9)
Age (years) Generation Z (18–27) Generation Y/Millennials (28–43) Generation X (44–59) Baby boomers (≥ 60)	110 (25.6) 176 (41.0) 95 (22.1) 48 (11.2)
Education No formal education Primary school Middle school High school Diploma Bachelor’s degree Master’s degree Doctorate degree	0 (0.0) 4 (0.9) 8 (1.9) 84 (19.6) 59 (13.8) 239 (55.7) 31 (7.2) 4 (0.9)
Occupation Student Governmental sector Private sector Freelancer Retired Unemployed	34 (7.9) 200 (46.6) 61 (14.2) 20 (4.7) 47 (11.0) 67 (15.6)
Marital status Married Single Divorced	294 (68.5) 114 (26.6) 21 (4.9)
Study major Medicine and Healthcare STEM (Science, Technology, Engineering, and Mathematics) Business and Administration/Law and Legal Studies Arts and Creative Industries Humanities and Social Sciences Non-profit and Social Services Others	48 (11.2) 44 (10.3) 64 (14.9) 8 (1.9) 30 (7.0) 7 (1.6) 228 (53.1)
	

**Fig 1:**
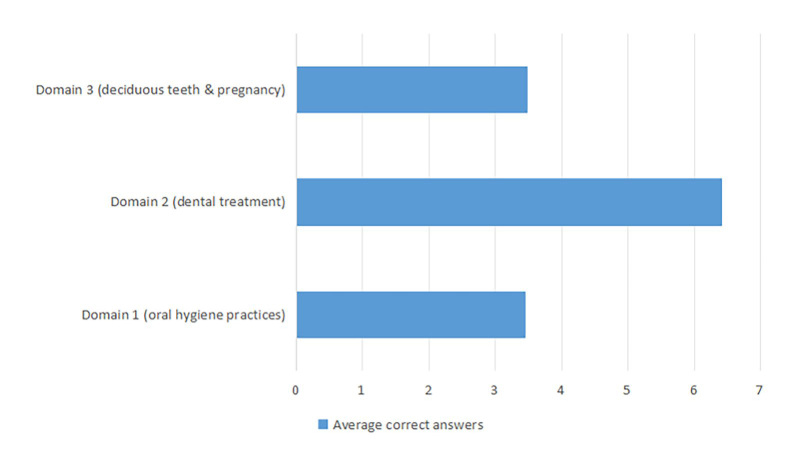
Average knowledge of the study participants regarding common dental myths in different domains.

The study assessed the participants’ knowledge of common dental myths across three domains: Domain 1, ‘Oral Hygiene Practices’; Domain 2, ‘Dental Treatment’; and Domain 3, ‘Deciduous Teeth and Pregnancy’ (Table 2). In the first domain, a high percentage of participants correctly identified myths, such as the ineffectiveness of hard bristles (77.6%) and the necessity of using a toothbrush and floss alongside Miswak (77.4%). However, less than half (41.3%) were aware that brushing should continue even if the gingiva bleeds. Similarly, almost half of them (54.5%) understood that products such as coal or salt do not clean teeth better. In the ‘Dental Treatment’ domain, many participants correctly understood that candies are not the only cause of tooth caries (89.0%), that tooth extraction is not always the best treatment (88.3%), and that regular dental visits are necessary even without pain (87.4%). However, the effects of misconceptions such as the impact of maxillary tooth extraction on eyesight (35.2%) and the brain (47.6%), as well as the use of painkiller tablets or other substances on painful teeth to relieve pain (41.7%), are less understood. Regarding the ‘deciduous teeth and pregnancy’ domain, most participants were aware that deciduous teeth need care (86.7%) and that a baby born with a tooth is not a sign of bad luck in the family (73.0%). However, myths such as teething cause fever (4.2%), and the baby absorbing calcium from the mother’s teeth during pregnancy (8.6%) is less well known. Figure 1 highlights the average knowledge of the study participants regarding common dental myths across the three different domains. The participants had the highest average knowledge (6.42) in the ‘Dental Treatment’ domain and relatively lower knowledge in the other two domains (Oral Hygiene Practices: 3.46; Deciduous Teeth and Pregnancy: 3.48). These findings highlight the significant gaps in knowledge regarding dental myths, indicating the need for targeted educational interventions.

**Table 2:** Knowledge of the participants regarding common dental myths in different domains (N = 429)

No	Item	Correct	(%)
Domain 1: Common myths regarding oral hygiene practices
1	Brushing the teeth with hard bristles make them whiter	333	77.6
2	Using Miswak only is enough, and there is no need for using the toothbrush, paste and floss	332	77.4
3	It is better not to brush the teeth when there is bleeding from the gums	177	41.3
4	Using products (eg, coal, salt) during brushing can clean the teeth better or make them whiter	234	54.5
5	A toothbrush can be shared among the same family members	409	95.3
Domain 2: Common myths regarding dental treatment
6	Removal of calculus lead to loosening of teeth and will weaken the tooth structure	286	66.7
7	The best treatment for a painful tooth is extraction	379	88.3
8	If you have pain on a particular tooth, placing a painkiller tablet or other substance on that tooth will reduce the pain	179	41.7
9	You do not need to visit the dentist if there is no pain in the teeth	375	87.4
10	Extraction of maxillary teeth will affect your eyesight	151	35.2
11	Extraction of maxillary teeth will affect your brain	204	47.6
12	Extracted permanent teeth do not need to be replaced with an artificial tooth	265	61.8
13	You should not eat anything when you are going for tooth extraction	229	53.4
14	Candies are the only cause of tooth decay	382	89.0
15	Tooth decay is a hereditary disease	305	71.1
Domain 3: Common myths regarding deciduous teeth and pregnancy
16	No need to take care of baby teeth because they will fall any way	372	86.7
17	Placing a milk bottle inside the baby’s mouth during sleep does not harm teeth	297	69.2
18	Teething causes fever (elevated body temperature)	18	4.2
19	During pregnancy, the baby absorbs calcium from the teeth and bones of his mother	37	8.6
20	With each pregnancy, the mother must lose a tooth	275	64.1
21	A baby born with a tooth is a sign of bad luck in the family	313	73.0
22	Pregnant ladies are not supposed to receive any dental treatment during pregnancy, and the treatment should be delayed until after delivery	181	42.2


The relationships between demographic factors and participants’ knowledge of common dental myths in the three different domains are presented in Table 3. Findings from the ‘Oral Hygiene Practices’ domain revealed significant associations with age, sex, education, occupation, and study major. On average, highly educated individuals had 0.31 points more education than did those with a high school degree or less (P value = 0.044). Compared with unemployed participants, freelancers had significantly greater average knowledge (0.90 points) (P value = 0.003). Those who studied ‘Medicine and Healthcare’ had 0.75% greater understanding than those who studied other majors (P value = 0.001). On the other hand, both sex and age were found to be associated with lower average knowledge regarding oral hygiene practices. Specifically, compared with female participants, males had 0.53% less knowledge compared to female participants (P value = 0.001), while those in Generation X (aged 44–59 years) had 0.42% less average knowledge than those in Generation Z (aged 18–27 years). However, the effect of age was borderline significant (P value = 0.077).

**Table 3:** Associations between demographic parameters and the knowledge regarding common dental myths in different domains (N = 429)

Parameter	Domain 1 (oral hygiene practices)	Domain 2 (dental treatment)	Domain 3 (deciduous teeth and pregnancy)	All domains
β coefficient (95% CI)	P value	β coefficient (95% CI)	P value	β coefficient (95% CI)	P value	β coefficient (95% CI)	P value
Sex (female)	Reference	Reference	Reference	Reference	Reference	Reference	Reference	Reference
Sex (male)	–0.53 (–0.78–0.27)	0.001 ^*^	0.10 (–0.32–0.53)	0.634	–0.55 (–0.86–0.24)	0.001 ^*^	–0.98 (–1.73–0.22)	0.011 ^*^
Age (Generation Z)	Reference	Reference	Reference	Reference	Reference	Reference	Reference	Reference
Age (Generation Y/Millennials)	–0.20 (–0.60–0.20	0.333	0.07 (–0.61–0.76)	0.824	0.08 (–0.47–0.58)	0.738	–0.03 (–1.52–1.17)	0.952
Age (Generation X)	–0.42 (–0.89–0.04)	0.077	0.57 (–0.22–1.37)	0.158	0.52 (–0.57–1.11)	0.077	0.67 (–0.73–2.09)	0.347
Age (Baby boomers)	–0.13 (–0.75–0.48)	0.674	0.73 (–0.31–1.79)	0.171	0.77 (0.04–1.54)	0.049 ^*^	1.37 (–0.48–3.23)	0.147
Education (high school or less)	Reference	Reference	Reference	Reference	Reference	Reference	Reference	Reference
Education (more than high school)	0.31 (0.01–0.62)	0.044 ^*^	0.58 (0.05–1.10)	0.031 ^*^	0.31 (–0.07–0.69)	0.113	1.20 (0.28–2.13)	0.011 ^*^
Occupation (unemployed)	Reference	Reference	Reference	Reference	Reference	Reference	Reference	Reference
Occupation (student)	–0.08 (–0.64–0.47)	0.764	–0.89 (–1.83–0.05)	0.063	–0.09 (–0.77–0.59)	0.796	–1.06 (–2.72–0.59)	0.208
Occupation (governmental)	0.24 (–0.12–0.62)	0.190	–0.11 (–0.74–0.51)	0.721	0.03 (–0.42–0.50)	0.870	0.17 (–0.94–1.28)	0.762
Occupation (private sector)	–0.01 (–0.44–0.42)	0.963	–0.45 (–1.19–0.27)	0.220	–0.01 (–0.54–0.52)	0.977	–0.47 (–1.76–0.81)	0.469
Occupation (freelancer)	0.90 (0.31–1.50)	0.003 ^*^	–0.15 (–1.16–0.85)	0.760	0.17 (–0.56–0.90)	0.651	0.92 (–0.86–2.70)	0.311
Occupation (retired)	0.39 (–0.20–0.98)	0.196	–0.35 (–1.36–0.65)	0.491	–0.43 (–1.17–0.29)	0.242	–0.40 (–2.18–1.38)	0.659
Marital status (married)	Reference	Reference	Reference	Reference	Reference	Reference	Reference	Reference
Marital status (single)	–0.29 (–0.63–0.07)	0.119	–0.07 (–0.70–0.56)	0.819	–0.62 (–1.08–0.15)	0.009 ^*^	–0.99 (–2.11–0.12)	0.083
Marital status (divorced)	–0.22 (–0.76–0.31)	0.416	0.45 (–0.46–1.36)	0.332	0.07 (–0.59–0.74)	0.827	0.30 (–1.30–1.91)	0.713
Study major (others)	Reference	Reference	Reference	Reference	Reference	Reference	Reference	Reference
Study major (medicine and healthcare)	0.75 (0.35–1.15)	0.001 ^*^	1.32 (0.64–2.00)	0.001 ^*^	1.18 (0.69–1.67)	0.001 ^*^	3.26 (2.06–4.45)	0.001 ^*^
Study major (STEM)	0.28 (–0.11–0.67)	0.165	0.32 (–0.35–0.99)	0.348	1.01 (0.52–1.50)	0.001 ^*^	1.61 (0.42–2.79)	0.008 ^*^
Study major (business and administration/law and legal studies)	0.19 (–0.14–0.53)	0.262	–0.17 (–0.75–0.40)	0.554	0.32 (–0.09–0.75)	0.128	0.34 (–0.67–1.37)	0.503
Study major (arts and creative industries)	0.11 (–0.71–0.95)	0.782	–0.46 (–1.88–0.94)	0.518	0.75 (–0.27–1.78)	0.152	0.40 (–2.09–2.90)	0.750
Study major (humanities and social sciences)	–0.15 (–0.61–0.30)	0.509	–0.04 (–0.83–0.73)	0.904	0.27 (–0.29–0.85)	0.339	0.07 (–1.31–1.45)	0.916
Study major (non-profit and social services)	–0.10 (–0.91–0.89)	0.982	0.34 (–1.19–1.87)	0.665	0.97 (–0.14–2.08)	0.087	1.30 (–1.38–4.00)	0.340
* P value ≤ 0.05 was considered statistically significant.

Both educational level and study major were found to statistically significantly impact participants’ knowledge regarding dental treatment. The average understanding of highly educated individuals was 0.58 points greater than that of less educated individuals (P value = 0.031). Similarly, those who studied ‘Medicine and Healthcare’ had 1.32% greater knowledge than those who studied other majors (P value = 0.001). However, occupation was associated with lower average knowledge. Specifically, individuals who were ‘students’ had 0.89% less knowledge regarding dental treatment myths than those who were ‘unemployed’. Nevertheless, the effect was of borderline significance (P value = 0.063).

For myths related to deciduous teeth and pregnancy, individuals who were 60 years or older (baby boomers) had 0.77% greater average knowledge than younger individuals (generation Z aged 18–27 years) (P value = 0.049). Additionally, those who were 44–59 years old (Generation X) had 0.52% greater average knowledge; however, this finding was borderline significant (P value = 0.077). Furthermore, a 1.18-point increase in average knowledge was observed among those who majored in Medicine and Healthcare (p value = 0.001), those who majored in Science, Technology, Engineering, and Mathematics or STEM) by 1.01 points (P value = 0.001), and those who majored in Non-profit and Social Services by 0.97. However, the effect for the latter was borderline statistically significantly (P value = 0.087). In contrast, sex and marital status were associated with lower average knowledge. Specifically, males had 0.55 lower average knowledge than females (P value = 0.001), while participants who reported being ‘single’ had 0.62 lower knowledge than those who were ‘married’ (P value = 0.009).

Regarding overall knowledge in all three domains, highly educated participants had an average level of knowledge that was 1.20 points greater than that of participants who were less educated (P value = 0.011). Moreover, those who majored in ‘Medicine and Healthcare’ had 3.26 points greater average knowledge (P value = 0.001), as did those who majored in STEM (1.61 points greater; P value = 0.008). In contrast, male participants and those who reported being ‘single’ showed lower overall knowledge across all three domains (0.98 points lower, P value = 0.011; 0.99 points lower, P value = 0.083, respectively). However, the effect of marital status was borderline significant.

## DISCUSSION

This study aimed to explore the knowledge of dental myths among participants in Taif city across different demographic groups. The findings underscore several noteworthy trends and associations, shedding light on the gaps in understanding and the potential impact of demographic factors on dental health education.

### Prevalence of Dental Myth

The study included 429 participants, predominantly female (53%), with Generation Y/Millennials (41.0%) and Generation Z (25.6%) being the majority age groups. Notably, 55.7% held a bachelor’s degree, indicating that having a well-educated sample likely influenced their dental health knowledge, and 68.5% were married, potentially impacting their health behaviours and knowledge. The study evaluated knowledge across three domains: oral hygiene practices, dental treatment, deciduous teeth and pregnancy. Participants demonstrated varying levels of understanding across these domains, with the highest average knowledge observed in the dental treatment domain.

In the domain of oral hygiene practices, most participants correctly identified some myths, such as the ineffectiveness of hard bristles and the necessity of using a toothbrush and floss alongside Miswak. These findings align with other studies, which found that the majority of respondents did not believe that brushing with hard bristles makes teeth whiter^3,13,14,15^ and that approximately half of the respondents disagreed that using Miswak alone is sufficient without a toothbrush and paste.^3,19^ Gaps were evident in areas such as the need to continue brushing despite bleeding gums and the misconception that products such as coal or salt clean teeth are better. This finding aligns with other studies,^2,3^ although another study by Salam et al (2023) found this finding to be less prevalent than that of the current study.^19^

Similarly, in the dental treatment domain, there was a high level of awareness regarding several myths. Participants correctly understood that candies are not the sole cause of tooth caries, consistent with findings from a study by Al Harthi (2019).^3^ They also recognised that tooth extraction is not always the best treatment option, in agreement with other studies.^3,6,14^ Additionally, the importance of regular dental visits, even in the absence of pain, was well acknowledged, contrasting with the findings of a study by Ain 2016.^2^ However, persistent misconceptions included beliefs about the effects of tooth extraction on eyesight or brain function and the belief that placing a painkiller tablet, or other substances can alleviate dental pain. These findings were consistent with those of Gambhir’s study (2015)^12^ but differed from those of Gowdar’s study (2021), which reported a lower prevalence of these myths.^13^

In the Deciduous Teeth and Pregnancy domain, myths such as deciduous teeth not needing care and a baby born with a tooth indicating bad luck were largely refuted, consistent with findings from Gowdar (2021).^13^ However, persistent myths included beliefs that teething causes fever, similar to Ain’s findings in 2016,^2^ and misunderstandings about babies absorbing calcium from the mother’s teeth during pregnancy, as documented by Al Harthi in 2019.^3^

#### Demographic Associations

The present study revealed several demographic factors significantly associated with participants’ knowledge of dental myths. Education emerged as a strong predictor across all domains, with higher educational level consistently linked to better understanding of oral health. Similarly, occupation played a significant role, particularly among freelancers and those in healthcare-related fields, who exhibited higher levels of knowledge across various domains. Freelancers exhibited greater knowledge of oral hygiene practices, possibly due to greater access to various information sources. Those with backgrounds in the Medicine and Healthcare or STEM fields showed notably greater knowledge, emphasising the positive impact of specialised education. This finding aligns with the literature,^12,13,19^ emphasising the role of education in health literacy and highlighting the need for tailored educational strategies for those with lower educational backgrounds.

Age and gender were also found to influence knowledge levels, albeit with varying impacts across different domains. Younger participants generally displayed greater awareness of oral hygiene practices, likely due to increased access to contemporary educational resources and digital media. ^6,12^ In contrast, older participants exhibited greater knowledge of myths related to deciduous teeth and pregnancy, possibly reflecting their broader life experiences and greater exposure to traditional health practices.^22^ Gender differences indicated that females tended to possess more comprehensive knowledge across all domains than males. This disparity could be attributed to differences in health-seeking behaviours, as females often engage more actively in preventive health measures and utilise healthcare services more frequently.^8,12,13,21^ Societal norms and roles may also play a crucial role, as women are typically more involved in caregiving and child-rearing, thereby acquiring more health-related information.

Marital status statistically significantly influenced knowledge about myths related to deciduous teeth and pregnancy. Married individuals demonstrated greater awareness in these areas,^9,10,16^ likely due to their increased exposure to childcare responsibilities and related health information.^23^ This group often interacts more frequently with healthcare professionals and participates in parental education programmes, thereby providing a better understanding of paediatric and maternal health. Conversely, single individuals exhibited lower overall knowledge, as they might not encounter these topics as part of their daily lives.

This study identified significant gaps in dental health knowledge, especially regarding myths about oral hygiene practices and dental care during pregnancy. Effective educational programmes should be tailored to specific demographic characteristics to ensure relevance and accessibility across diverse populations. Integrating dental health education into broader health promotion initiatives, particularly for non-healthcare professionals, could substantially improve overall public awareness. Future research should focus on evaluating the effectiveness of different educational strategies and interventions tailored to demographic factors to achieve widespread and equitable improvements in dental health knowledge.

This study has limitations, including its reliance on self-reported data and its cross-sectional design, which limits causal inference. The specific cultural context of Taif city may also affect generalizability. Future research should consider longitudinal and intervention studies, broader and more diverse samples, qualitative methods, and comparative studies across regions.

## CONCLUSION

This study provides valuable insights into the prevalence of dental myths in Taif city and their associations with demographic factors. While higher education levels and specific fields of study correlate with better knowledge, substantialt gaps persist across different demographic groups. This study underscores the importance of targeted educational interventions to address dental myths, especially among less educated individuals, certain occupational groups, and specific demographic segments such as males and single individuals. Addressing these disparities through focused public health initiatives has the potential to enhance dental health knowledge and behaviours in Taif city, thereby fostering improved oral health outcomes across the community.

### Acknowledgements

The researcher would like to acknowledge the Deanship of Scientific Research at Taif University for funding this work.
